# Comment on Chen et al. Vestibulo-Ocular Reflex Results in Patients with Intralabyrinthine Schwannomas: Case Series with a Literature Review. *Diagnostics* 2025, *15*, 2093

**DOI:** 10.3390/diagnostics16101535

**Published:** 2026-05-19

**Authors:** Eugen Constant Ionescu

**Affiliations:** Hôpital Edouard Herriot, 69003 Lyon, France; eugen.ionescu@chu-lyon.fr

We read with great interest the recent article by Chen et al. on vestibulo-ocular reflex findings in intralabyrinthine schwannoma (ILS) [1]. The topic is clinically important, especially because ILS may overlap phenomenologically with Ménière-like audiovestibular syndromes and other retrocochlear disorders. We would like to highlight several points that may further refine the interpretation of the authors’ cases and their methodological approach.

First, based on the MRI figures provided in the article, several cases—particularly patients 2, 3, and 4—appear to show a relatively reduced internal auditory canal (IAC) caliber. This raises the possibility of a concomitant narrowed or near-narrowed IAC, a condition that has been described as a distinct clinic radiological entity associated with cochleovestibular symptoms, including vertigo, dizziness, and hearing loss [2,3]. Recent work has emphasized that narrowed IAC morphology may be under-recognized unless the canal is specifically assessed, ideally in the coronal plane [3]. In our view, this point deserves consideration because reduced IAC dimensions may constitute a confounding anatomical factor when symptoms are attributed solely to an intralabyrinthine lesion.

Second, the interpretation of vestibular symptoms in ILS should remain cautious. While recurrent vestibular attacks have been described in intravestibular schwannomas and may occasionally dominate the clinical presentation, the available literature suggests that they do not constitute the predominant phenotype across reported series, which remain heterogeneous and primarily driven by auditory symptoms [4,5,6]. By contrast, intracochlear schwannomas tend to present mainly with auditory dysfunction, often severe from onset, with limited vestibular involvement unless there is extension beyond the cochlea [5]. Likewise, Ménière-like presentations have been reported in classical vestibular schwannoma, but they appear to represent a minority pattern rather than the dominant clinical phenotype [7]. For this reason, in patients with apparent intravestibular tumors and recurrent vestibular symptoms, it may be difficult to determine how much of the symptom burden is directly tumor-related and how much may be modulated by associated canal morphology or other retrocochlear mechanisms.

Third, from a methodological perspective, we noted that the hyperventilation test was not included among the vestibular assessments. This is potentially relevant because hyperventilation-induced nystagmus has repeatedly been reported as a useful marker of eighth cranial nerve-type dysfunction, including vestibular schwannoma [8,9,10,11]. Minor et al. demonstrated hyperventilation-induced nystagmus in patients with vestibular schwannoma, and later studies by Choi et al., Mandalà et al., and Califano et al. further supported its diagnostic value in vestibular schwannoma and related retrocochlear settings [8,9,10,11]. Although this test cannot distinguish between a tumoral retrocochlear process and alternative mechanisms such as focal neuropathy, local compression within a narrowed IAC, or even a neurovascular conflict favored by a small canal, it could nevertheless provide useful complementary functional information.

This issue may be particularly pertinent in the authors’ patients 2–4, where the IAC appears relatively small on the published images. In such cases, the coexistence of ILS with narrow IAC anatomy could create diagnostic ambiguity and potentially lead to over-attribution of vestibular manifestations to the intralabyrinthine tumor itself.

We therefore suggest that future studies of ILS systematically evaluate IAC morphology, preferably with dedicated coronal reconstructions, and consider including the hyperventilation test as an adjunctive bedside assessment of retrocochlear involvement. We believe that integrating these anatomical and functional parameters may improve diagnostic precision in patients presenting with Ménière-like and/or stereotyped audiovestibular syndromes.
